# Controlling DNA-End Resection: An Emerging Task for Ubiquitin and SUMO

**DOI:** 10.3389/fgene.2016.00152

**Published:** 2016-08-23

**Authors:** Sarah-Felicitas Himmels, Alessandro A. Sartori

**Affiliations:** Institute of Molecular Cancer Research, University of ZurichZurich, Switzerland

**Keywords:** DNA double-strand break repair, DNA-end resection, homologous recombination, ubiquitylation, sumoylation, CtIP/Sae2

## Abstract

DNA double-strand breaks (DSBs) are one of the most detrimental lesions, as their incorrect or incomplete repair can lead to genomic instability, a hallmark of cancer. Cells have evolved two major competing DSB repair mechanisms: Homologous recombination (HR) and non-homologous end joining (NHEJ). HR is initiated by DNA-end resection, an evolutionarily conserved process that generates stretches of single-stranded DNA tails that are no longer substrates for religation by the NHEJ machinery. Ubiquitylation and sumoylation, the covalent attachment of ubiquitin and SUMO moieties to target proteins, play multifaceted roles in DNA damage signaling and have been shown to regulate HR and NHEJ, thus ensuring appropriate DSB repair. Here, we give a comprehensive overview about the current knowledge of how ubiquitylation and sumoylation control DSB repair by modulating the DNA-end resection machinery.

## Introduction

The capacity of our cells to detect and repair damaged DNA is key to prevent genomic instability and consequently the development of cancer (Jackson and Bartek, 2009; Ciccia and Elledge, 2010). DNA double-strand breaks (DSBs) are particularly hazardous lesions as their inappropriate repair can result in chromosomal translocations, an important driving force of tumorigenesis (Hanahan and Weinberg, 2011; Forment et al., 2012; Bunting and Nussenzweig, 2013; Rodgers and McVey, 2016). To circumvent this threat, the balance between the two major DSB repair pathways – homologous recombination (HR) and classical non-homologous end-joining (C-NHEJ) – is governed by various factors (Chapman et al., 2012; Ceccaldi et al., 2016). C-NHEJ operates with fast kinetics throughout the entire cell cycle and directly ligates broken DNA ends without requiring extended sequence complementarities to guide repair (Chiruvella et al., 2013; Radhakrishnan et al., 2014; Graham et al., 2016). In contrast, HR is slower and restricted to the S and G2 phases of the cell cycle because it requires an intact sister chromatid as a template for homology-directed repair (Karanam et al., 2012; Jasin and Rothstein, 2013; Orthwein et al., 2015). HR is initiated by DNA-end resection, an evolutionarily conserved mechanism that generates long stretches of 3′ single-stranded DNA (ssDNA) overhangs by nucleolytic degradation of the 5′ terminated strand of the DSB (Symington, 2014; Cejka, 2015; Daley et al., 2015). Consequently, DNA-end resection is a prerequisite for the formation of the Rad51-ssDNA presynaptic filament to promote HR. At the same time, it precludes the assembly of the C-NHEJ machinery, most prominently the Ku70-Ku80 (Ku) heterodimer, to bridge and ligate the broken DNA ends (Symington and Gautier, 2011). Thus, being a critical determinant of DSB repair pathway choice, DNA-end resection is tightly controlled through multiple mechanisms, including post-translational modifications (PTMs). For instance, core components of the DSB resection machinery as well as resection antagonists undergo phosphorylation by cyclin-dependent kinases to gradually shift DSB repair from NHEJ to HR in the postreplicative stages of the cell cycle (Ferretti et al., 2013; Tomimatsu et al., 2014; Tkáč et al., 2016). In addition to phosphorylation, recent evidence highlighted that ubiquitylation and sumoylation control almost every aspect of cellular responses to DNA damage, including the repair of DSBs (Jackson and Durocher, 2013; Schwertman et al., 2016). This was exemplified by high-throughput proteomics studies revealing that DSB repair is facilitated by waves of global DNA damage-induced ubiquitylation and sumoylation (Cremona et al., 2012; Psakhye and Jentsch, 2012; Elia et al., 2015).

Ubiquitin and small ubiquitin-related modifier (SUMO), the most prominent members of a conserved protein family of ubiquitin-like proteins, can be attached to lysine residues of target proteins *via* an isopeptide bond (Bergink and Jentsch, 2009). There is only one SUMO in yeast (encoded by the essential *smt3* gene), whereas vertebrates express three independent SUMO isoforms (SUMO-1,-2,-3), of which SUMO-2/3 share 97% sequence identity (Hay, 2013). Different from other PTMs, ubiquitin-like modifications are carried out in a three-step cascade mechanism requiring the consecutive action of activating enzymes (E1s), conjugating enzymes (E2s), and ligases (E3s), which confer substrate specificity. In humans, ubiquitylation is mediated by two E1s, ∼35 active E2s, and more than 600 E3s, while sumoylation is conducted by a single heterodimeric E1, one E2 (UBC9), and approximately 10 E3s (Komander and Rape, 2012; Flotho and Melchior, 2013; Berndsen and Wolberger, 2014; Brown and Jackson, 2015; Stewart et al., 2016). Both processes are reversible with the removal of ubiquitin and SUMO from substrate proteins performed by deubiquitinases (DUBs) and SUMO/sentrin-specific proteases (SENPs), respectively (Ronau et al., 2016). Ubiquitin can be attached to target proteins either as monoubiquitin or as different types of polyubiquitin chains, depending on which of the seven lysine residues of ubiquitin is used for chain assembly (Swatek and Komander, 2016; Yau and Rape, 2016). The diverse ubiquitin chain types having different structural properties can change a variety of attributes in the target proteins. For example, while K48-linked ubiquitin chains promote proteasomal degradation, K63-linked chains are generally considered to regulate protein-protein interactions. In contrast, poly-SUMO chains primarily form through a single consensus sumoylation motif in mammalian SUMO-2/3, which is missing in SUMO-1 (Hay, 2013).

In this review, we want to highlight the importance of ubiquitin and SUMO in DSB repair with a special focus on the regulation of DNA-end resection.

## DNA-End Resection in a Nutshell

**FIGURE 1:**
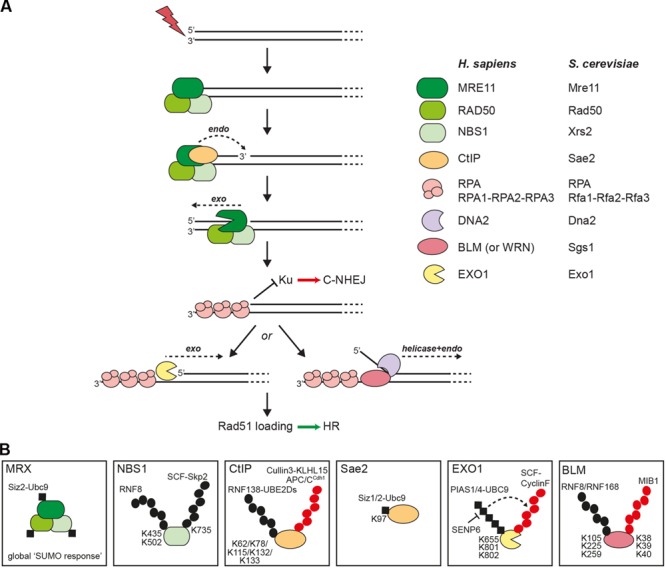
**DNA-end resection factors are modified by ubiquitin and SUMO. (A)** Simplified scheme of the bidirectional DNA-end resection model. Upon DSB induction the MRX/N complex rapidly localizes to the damaged site. During S and G2 phases of the cell cycle, DNA-end resection is needed for the repair of DSBs via homologous recombination (HR). According to the newest biochemical evidence in yeast, MRX and Sae2 collaborate in the initiation of DNA-end resection through endonucleolytic cleavage of the 5′-terminated strand upstream from the DSB end. Starting from the nick, the exonuclease activity of Mre11 is then supposed to degrade DNA in a 3′ to 5′ direction back toward the DSB end. The resulting single-stranded DNA (ssDNA) overhang is immediately coated by RPA to protect the ssDNA from degradation. The 5′-recessed end now represents a preferred substrate for the 5′ to 3′ exonuclease Exo1 to carry out more processive resection. Alternatively, extended resection is catalyzed by the combined endonuclease and helicase activities of Dna2-Sgs1 in yeast or DNA2-BLM (or WRN) in human cells. Importantly, processed DSB ends are no longer a substrate for Ku binding, a prerequisite for DSB repair by classical non-homologous end joining (C-NHEJ). Ultimately, RPA is removed from ssDNA and replaced by the Rad51 recombinase to initiate strand invasion of the sister chromatid and further downstream steps in HR. **(B)** Schematic illustration of selected resection factors undergoing ubiquitylation and/or sumoylation. Please refer to the main text for details. Black dots, ubiquitin modifications involved in modulating protein function; red dots, ubiquitin modifications involved in protein degradation; black squares, SUMO modification; K, ubiquitin- or SUMO-modified lysine residues in substrate proteins.

DNA-end resection in eukaryotes is a bidirectional two-step process initiated by the MRX (Mre11-Rad50-Xrs2) nuclease complex in conjunction with Sae2 in yeast, and by the MRN (MRE11-RAD50-NBS1) complex in conjunction with CtIP in human cells (**Figure 1A**). Subsequently, extended resection is performed by two redundant mechanisms involving either the 5′ to 3′ exonuclease Exo1 or the endonuclease Dna2 in concert with the RecQ helicase Sgs1 in yeast, and either EXO1 or DNA2 in concert with BLM (or WRN) in human cells (**Figure 1A**) (Sturzenegger et al., 2014; Cejka, 2015; Symington, 2016). As a result of this process, stretches of ssDNA are rapidly coated by RPA, the heterotrimeric ssDNA-binding protein, which serves as a platform to activate cell cycle checkpoints. For the ssDNA to be used as a substrate for homology-directed repair, RPA needs to be replaced by Rad51 with the help of recombination mediators (e.g., BRCA2).

## Ubiquitylation and Sumoylation of the DNA-End Resection Machinery

### MRN/MRX Nuclease Complex

Mass spectrometric analysis revealed several potential ubiquitylation sites in all three subunits of the MRN complex (Kim et al., 2011; Symington and Gautier, 2011; Wagner et al., 2011; Mertins et al., 2013; Elia et al., 2015). However, with the exception of NBS1, none of them have yet been experimentally validated. Skp2, an F-box protein and component of the SCF (Skp1-Cullin1-F-box) E3 ligase complex, was found to interact with NBS1 and conjugate K63-linked ubiquitin chains onto NBS1-K735 in response to DSBs (**Figure 1B**) (Wu et al., 2012). Although DNA-end resection was not investigated in this study, cells deficient for Skp2 exhibited defects in ATM activation and HR (Wu et al., 2012). Lu et al. (2012) reported that RNF8 ubiquitinates NBS1 at two lysine residues and this was further shown to promote NBS1 recruitment to laser-induced DSB sites. Interestingly, cells ectopically expressing the ubiquitylation-deficient mutant of NBS1 exhibited reduced RPA foci formation after IR treatment and decreased HR frequency (**Figure 1B**) (Lu et al., 2012).

It has been discovered through proteomics studies that DNA damage-induced multisite sumoylation of a subset of HR proteins in yeast, including MRX, accelerates DSB repair and that this global ‘SUMO response’ depends on both MRX and DNA-end resection (**Figure 1B**) (Cremona et al., 2012; Psakhye and Jentsch, 2012). Consistently, Psakhye and Jentsch (2012) reported that *S. cerevisiae* Mre11 is sumoylated and exhibits strong two-hybrid interactions with Ubc9 (E2) and Siz2 (E3). However, very recent findings suggested that sumoylation of Mre11 is unlikely to be required for MRX-dependent DNA-end resection but that SUMO-interacting motifs (SIMs) in Mre11 non-covalently recruit poly-SUMO chains to facilitate MRX complex assembly (Chen et al., 2016).

### CtIP/Sae2

Several E3 ubiquitin ligases have been described to interact with and modify CtIP, thereby possibly affecting DNA-end resection and DSB repair pathway choice. An early study reported that the heterodimeric RING-type E3 ligase BRCA1/BARD1 ubiquitylates CtIP to promote its stable retention at sites of DNA damage (Yu et al., 2006). However, more recent data indicated that BRCA1 specifically ubiquitylates histone H2A, thereby rendering the chromatin permissive for long-range resection after initial resection by CtIP-MRN has occurred (Kalb et al., 2014; Densham et al., 2016). Moreover, Schmidt et al. (2015) demonstrated that RNF138 in complex with the UBE2D family of E2 conjugating enzymes interacts with CtIP to foster its ubiquitylation and accumulation at DSBs (**Figure 1B**). The authors further observed that depletion of pivotal RING-type E3 ligases involved in the DDR including BRCA1, RNF8 and RNF168 does not compromise DNA damage accrual of CtIP (Schmidt et al., 2015). Therefore, the physiological role of BRCA1-dependent CtIP ubiquitylation in DNA repair still remains to be determined (Barber and Boulton, 2006). Mass spectrometry analysis of CtIP from irradiated cells revealed 13 potential ubiquitylation sites (Schmidt et al., 2015). Furthermore, the same authors found that CtIP polyubiquitylation and redistribution to DSBs was impaired in cells expressing a CtIP mutant in which five N-terminal lysine residues were simultaneously substituted with arginines (5KR) (Schmidt et al., 2015). Finally, as ectopic expression of CtIP-5KR did not restore DNA-end resection in CtIP-depleted cells, it was proposed that ubiquitylation of CtIP by RNF138-UBE2D is a key event in promoting HR (Schmidt et al., 2015). Further support for a pro-resection function of RNF138 emerged from another study showing that RNF138 ubiquitylates Ku80 to facilitate the removal of Ku from DSBs, thereby allowing access of the DNA-end resection machinery and subsequent HR (Ismail et al., 2015). Taken together, one could envision that RNF138-mediated CtIP recruitment to, and Ku displacement from DSBs act in parallel to promote DNA-end resection. Yet it may be possible that RNF138 targets additional proteins involved in DSB repair pathway choice (Bekker-Jensen and Mailand, 2015). Interestingly, two independent studies have reported that the deubiquitinase (DUB) activity of USP4 functions in DNA-end resection (Liu et al., 2015; Wijnhoven et al., 2015). They both demonstrated that USP4 interacts with CtIP and MRN and regulates the recruitment of CtIP to DSBs. However, they further observed that USP4 auto-deubiquitylation rather than USP4-mediated deubiquitylation of CtIP is essential for HR.

The anaphase-promoting complex/cyclosome-Cdh1 (APC/C^Cdh1^) E3 ubiquitin ligase was shown to control cell cycle-dependent repair of DSBs by specifically targeting CtIP for proteasomal degradation after mitotic exit as well as after DNA damage in G2 phase (**Figure 1B**) (Lafranchi et al., 2014). Conceivably, such a mechanism would counteract resection of DSBs and allow efficient C-NHEJ in G1 cells, where the intact sister chromatid is not available for HR. Consistently, it was demonstrated that expression of a CtIP mutant defective in Cdh1 interaction abolished CtIP ubiquitylation, leading to its accumulation and prolonged retention at DSBs, oversized DNA-end resection and impaired DSB repair (Lafranchi et al., 2014). Furthermore, a similar cell cycle-dependent mechanism resulting in ubiquitin-mediated proteolysis of CtIP was shown to involve the peptidyl-prolyl *cis*/*trans* isomerase PIN1 (Steger et al., 2013). Following DSB induction in G2, PIN1 was found to specifically interact with CtIP through two phosphorylated S/T-P motifs, leading to its ubiquitylation and subsequent proteasomal degradation (Steger et al., 2013). Consequently, the PIN1-CtIP axis was equally proposed to antagonize DNA-end resection, particularly in situations where NHEJ is the preferred pathway. Moreover, it has been suggested that PIN1-mediated CtIP isomerization triggers a conformational change which facilitates the binding of a E3 ubiquitin ligase (Sartori and Steger, 2013). Our most recent findings point toward a role for the Cullin3 (CUL3) E3 ubiquitin ligase in cooperating with PIN1 in the regulation of CtIP protein stability (**Figure 1B**) (Ferretti et al., 2016). In brief, we discovered that the CUL3 substrate adaptor Kelch-like protein 15 (KLHL15) interacts with CtIP to promote its degradation via the ubiquitin-proteasome pathway. Accordingly, we observed that DNA-end resection is strongly decreased in cells overexpressing KLHL15 but enhanced in cells lacking KLHL15, thus impacting the balance between HR and NHEJ.

Using reconstituted SUMO conjugating systems, both CtIP and Sae2 were found to be sumoylated (Sarangi et al., 2015). Moreover, Ubc9-Siz1/2-mediated Sae2 sumoylation at a single conserved lysine residue (K97) was induced by DNA damage and found to increase the levels of soluble Sae2 (**Figure 1B**). Further genetic analysis revealed that Sae2-K97R mutant cells are impaired in the processing and repair of DSBs, indicating that Sae2 sumoylation is critical for DNA-end resection.

In summary, ubiquitin and SUMO control CtIP/Sae2 resection function at various levels, including its redistribution at DSBs, protein-protein interactions and protein stability.

### EXO1 5′ to 3′ Exonuclease

It has been known for quite some time that human EXO1 is targeted for degradation by the ubiquitin-proteasome pathway in response to treatment with agents that block DNA replication (El-Shemerly et al., 2005, 2008). Interestingly, work from the same group could recently demonstrate that EXO1 is constitutively sumoylated by PIAS1/4-UBC9 *in vitro* and *in vivo* and that this is a prerequisite for ubiquitin-mediated EXO1 degradation at stalled replication forks avoiding excessive resection of free DNA ends (**Figure 1B**) (Bologna et al., 2015). Moreover, they found that the SENP6 de-sumoylating enzyme interacts with EXO1 to antagonize this process. However, since mutating three major SUMO acceptor sites in EXO1 did not effectively rescue EXO1 degradation it remains to be determined how, mechanistically, EXO1 sumoylation controls its enzymatic activity (Bologna et al., 2015). Consistent with these findings, Elia et al. (2015) reported that EXO1 is ubiquitylated and degraded by the proteasome in response to replication stress induced by UV radiation and 4NQO. They further identified EXO1 as a new substrate of the SCF-Cyclin F E3 ubiquitin ligase, which possibly mediates EXO1 degradation to prevent unwanted resection of stalled forks (Elia et al., 2015). Finally, adding another layer of complexity to the regulation of EXO1 by ubiquitin and SUMO, Nishi et al. (2014) recently discovered that the proteasome-associated DUB UCHL5 contributes to DNA-end resection, at least in part, by regulating the recruitment of EXO1 (but not CtIP) to sites of DNA damage.

### DNA2/Dna2 Structure-Specific Endonuclease

More than 20 potential ubiquitylation sites on human DNA2 have so far been identified in different mass spectrometry approaches, but their role in DNA damage/repair has not yet been experimentally addressed (Kim et al., 2011; Wagner et al., 2011; Mertins et al., 2013).

### BLM/Sgs1 3′ to 5′ DNA Helicase

Besides promoting long-range resection of DSBs in conjunction with DNA2, BLM has important functions in other DNA metabolic pathways including DNA replication, telomere maintenance and transcription (Croteau et al., 2014). BLM sumoylation and ubiquitylation has previously been proposed to control its spatiotemporal localization and to promote or suppress HR particularly in the context of stalled replication forks (Eladad et al., 2005; Ouyang et al., 2013; Tikoo et al., 2013). Akin to these observations, Sgs1 and BLM nuclear foci formation in response to hydroxyurea (HU) treatment was found to be negatively regulated by the SUMO-targeted ubiquitin ligase complexes Slx5-Slx8 and RNF4 in yeast and mammalian cells, respectively (Böhm et al., 2015). Moreover, Tikoo et al. (2013) reported that cells lacking either RNF8 or RNF168 E3 ligases failed to efficiently promote K63-linked ubiquitylation of BLM following HU exposure, which is otherwise required for BLM-RAP80 interaction and, thus, BLM recruitment to damaged chromatin (**Figure 1B**). A controversial issue relates to the question as to whether or not TOPBP1-BLM interaction, which is important for genome maintenance, protects BLM from MIB1-mediated ubiquitylation and subsequent proteasomal degradation when cells encounter DNA damage during S phase (**Figure 1B**) (Wang et al., 2013, 2015; Blackford et al., 2015).

Notably, following DNA-end resection, the RPA-ssDNA platform becomes extensively modified by ubiquitin and SUMO to promote checkpoint activation and HR in both yeast and human cells, as it has been recently reviewed elsewhere (Maréchal and Zou, 2015; Schwertman et al., 2016). Finally, emerging data from the Durocher lab demonstrates that ubiquitylation of PALB2, a major binding partner of BRCA2, by the E3 ligase CUL3^KEAP1^ blocks its interaction with BRCA1 and, consequently, the recruitment of BRCA2 to DSBs, thereby suppressing HR in G1 cells (Orthwein et al., 2015).

## Conclusion and Future Perspectives

The key discovery that E3 ubiquitin ligases RNF8 and RNF168 play an integral part in the crosstalk between chromatin state and DNA damage signaling has opened the door for scientists to investigate how ubiquitin and SUMO orchestrate DSB repair pathways. In the last few years, it became clear that RNF8-RNF168-mediated ubiquitylation of histones mainly serves to generate recruitment platforms for the coordinated assembly of various ubiquitin-binding domain (UBD)-containing proteins (e.g., 53BP1) to DSB sites (Schwertman et al., 2016). In contrast, ubiquitin-mediated recruitment seems to play a minor role in the regulation of DNA-end resection, which is further supported by the fact that resection factors are devoid of any canonical UBDs. Although Murina et al. (2014) reported that CtIP can interact with ubiquitin *in vitro*, further investigations are clearly needed to establish a role for CtIP-ubiquitin interaction in DNA-end resection.

Our survey revealed that ubiquitylation and sumoylation of DNA-end resection factors predominantly influences protein stability, thereby facilitating their timely removal to enable the completion of HR (**Table 1**). Another emerging theme is that ubiquitin-mediated proteolysis of resection proteins is dependent on the cell cycle stage and may therefore need to be primed by an upstream phosphorylation event. In other words, an important challenge for the future will be to investigate whether and how ubiquitin and SUMO are able to fine-tune nuclease and/or helicase activities of specific resection enzymes. Current evidence suggests that SUMO may preferentially function as an intermolecular ‘glue’ in modulating protein-protein or protein-DNA interactions required for HR rather than specifically affecting the activity of individual proteins (Sarangi and Zhao, 2015). Finally, there is only very limited data available yet regarding the role of deconjugating enzymes in DSB repair. As they belong to a family of cysteine proteases and are therefore considered more ‘druggable’ than E3 ligases, the identification of DUBs or SENPs promoting DNA-end resection and HR could provide a new basis for the development of inhibitors for targeted cancer therapy (Hühn et al., 2013; Carvalho and Kanaar, 2014; D’Arcy et al., 2015).

**Table 1:** DNA-end resection proteins targeted by ubiquitin or SUMO E3 ligases.

DNA-end resection factor	E3 Ligase	Modification	Reference
Mre11/Rad50/Xrs2	*global sumoylation response*	SUMO	Cremona et al., 2012; Psakhye and Jentsch, 2012
NBS1	RNF8	Ubiquitin	Lu et al., 2012
	SCF^Skp2^	Ubiquitin	Wu et al., 2012
CtIP	BRCA1/BARD1	Ubiquitin	Yu et al., 2006
	CUL3^KLHL15^	Ubiquitin	Ferretti et al., 2016
	APC/C^Cdh1^	Ubiquitin	Lafranchi et al., 2014
	RNF138	Ubiquitin	Schmidt et al., 2015
Sae2	Siz1/2	SUMO	Sarangi et al., 2015
BLM	RNF8 and RNF168	Ubiquitin	Tikoo et al., 2013
	MIB-1	Ubiquitin	Blackford et al., 2015; Wang et al., 2013
EXO1	PIAS1/4	SUMO	Bologna et al., 2015
	SCF^CyclinF^	Ubiquitin	Elia et al., 2015

## Author Contributions

All authors listed, have made substantial, direct, and intellectual contributions to the work, and approved it for publication.

## Conflict of Interest Statement

The authors declare that the research was conducted in the absence of any commercial or financial relationships that could be construed as a potential conflict of interest.

The reviewer WN and handling Editor declared their shared affiliation, and the handling Editor states that the process nevertheless met the standards of a fair and objective review.
